# Alkaline loading of extracellular vesicles produced from human neural stem cell-derived neurospheres enables CNS drug delivery

**DOI:** 10.21203/rs.3.rs-7622911/v1

**Published:** 2025-09-23

**Authors:** Amar M. Singh, Charles M. White, Adeline Phillips, Logan P. Crowe, Robert Marti, Morgan C. Finnerty, Martonio Ponte Viana, William Antoniades, Michael G. Bartlett, Viviana Martinez, Raymond Swetenburg, Steven L. Stice

**Affiliations:** University of Georgia; Aruna Bio, Inc; Aruna Bio, Inc; Aruna Bio, Inc; Aruna Bio, Inc; University of Georgia; Aruna Bio, Inc; Aruna Bio, Inc; University of Georgia; Aruna Bio, Inc; Aruna Bio, Inc; University of Georgia

**Keywords:** Extracellular vesicles, exosomes, doxorubicin, neural stem cells, neural progenitor cells, blood-brain barrier, glioblastoma, drug delivery, alkaline loading

## Abstract

The blood brain barrier and blood tumor barrier (BBB and BTB, respectively) represent significant obstacles for the delivery of drugs to treat diseases of the central nervous system, such as brain cancers and neurodegenerative diseases. Extracellular vesicles (EVs) or exosomes have emerged as a new drug delivery vehicle for CNS diseases as they may penetrate the BBB/BTB and are less immunogenic than liposomal carriers. EVs derived from human neural stem cells (hNSC) provide additional benefits over other EV sources due to their increased homing capability to neural cells and demonstrated efficacy for treating stroke and traumatic brain injury in rodent models. However, the utilization of EVs from hNSC for drug delivery remains largely unexplored, due in part to difficulties in manufacturing capacity compared to traditional cell lines. Here, we report the development of a hNSC suspension neurosphere system for EV production and drug delivery. As proof of concept, doxorubicin was loaded into hNSC-EV, using a novel, high-efficiency alkaline passive loading method, and shown to be effective at inducing cytotoxicity in glioma cells *in vitro* and exhibiting higher BBB penetrance than doxorubicin-alone *in vivo*. These studies demonstrate the potential for hNSC-EV loaded doxorubicin as a therapeutic treatment for brain cancers such as glioblastoma, while also establishing hNSC-EVs as a drug-delivery vehicle for CNS diseases.

## 1. Introduction

The blood-brain barrier (BBB) serves a critical function in protecting the brain and spinal cord from toxins and other chemical insults, while maintaining the necessary homeostatic balance to maintain oxygen and nutrient levels [1]. However, due to this strict control between the vasculature and the central nervous system (CNS), the delivery of therapeutics to treat CNS diseases is severely hampered [2]. Furthermore, upon the development of a primary brain tumor or brain metastasis from a non-CNS tumor, a blood-tumor barrier (BTB) is established. Like the BBB, the BTB serves a restrictive role in preventing the delivery of therapeutics, which could otherwise be used to treat and prevent further tumor progression [3].

Over the past decade, multiple approaches, including receptor-mediated transcytosis, nanoparticles and extracellular vesicles (EVs), have been evaluated to enable the delivery of drugs through the BBB/BTB to the CNS with varying levels of success [2, 4]. The method that has been most intensely investigated is the use of the Transferrin receptor (TfR), which relies on receptor-mediated transcytosis to transport a cargo through the BBB [4]. Several caveats to this approach exist however, such as lack of brain specificity [5], TfR recycling partially preventing exocytosis in the brain [6, 7], and lysosomal degradation [8]. Several other receptors are still being evaluated, with most still in the pre-clinical stage [2, 4]. Nanoparticle delivery, offer some utility for CNS drug delivery, as drugs can be loaded directly in a scalable manner and the nanoparticles can be chemically-modified to facilitate CNS-targeting [9]. Weaknesses with nanoparticles, however, such as rapid clearance, immune-tolerability, and the lack of an efficient, validated brain targeting method limit their current uses for CNS diseases. EVs, on the other hand, may offer several key advantages over nanoparticles for drug delivery through the BBB/BTB [10].

EVs, such as exosomes and microvesicles, have been reported to traverse the BBB through mechanisms that are still yet to be fully elucidated [11, 12]. Irrespective to the mechanism, EVs provide a significant opportunity for the delivery of cargo, including small molecules, RNA and protein, to the CNS for disease treatment. Indeed, numerous studies have described methods for loading EVs with various cargo using exogenous (e.g. sonication, electroporation, passive incubation) or endogenous (genetic modification of the EV-donor cell) methods [13]. There are several clinical trials that have been initiated or are currently ongoing evaluating the efficacy of drug-loaded EVs in non-CNS indications, especially for cancer treatments [14].

An important aspect of EV production for drug-delivery is the choice of donor cells. To date, most of the therapeutic development around EVs has focused on utilizing HEK293 cells or mesenchymal stem cells (MSC) as the donor cell type, as these cells are easy to grow in a high-production capacity. However, for treating CNS-diseases, these cells may not serve as an ideal choice as several studies have indicated that EVs possess natural homing capability to their tissue and/or cell-type of origin [15, 16]. For example, it has been demonstrated that EVs from lung, liver and brain tumor cells exhibit preferential uptake into their organ of origin [17]. Potentially, EVs from neural cell types, such as human neural stem cells (hNSC), may provide increased uptake into CNS cells compared to MSC as they more closely align with the cell of origin. In support of this, the therapeutic efficacy of hNSC-derived EVs outperformed MSC-EV in a murine embolic stroke model [18]. EVs from other neural cell types, such as astrocytes, have also been described [19]. One weakness with utilizing human astrocytes, either from primary or hPSC sources, versus hNSCs for EV production is that astrocytes are unable to self-renew and be cultured long-term [20, 21], and therefore have significant scalability constraints limiting their use for therapeutic development.

Human neural stem cells (hNSC) serve as a valuable donor cell type for EV production due to their self-renewal capability with moderate-to-high proliferation rate, karyotypic stability upon long-term passaging and neural targeting capabilities [22–27]. These neural EVs have been successfully validated as a therapeutic in animal models of stroke and traumatic brain injury [18, 28–31], and may therefore provide added benefits for some CNS diseases. Further, cell aggregates in suspension have been scaled in large cell bioreactors [32–34]. Therefore, we expanded the potential of hNSC-derived EVs by establishing a hNSC neurosphere culture system for EV collection and demonstrated their drug-delivery potential by loading the EVs with doxorubicin to evaluate brain uptake levels *in vivo*. To this end, we successfully developed a novel alkaline passive loading methodology that proved to be highly effective at Dox incorporation into EVs. Overall, the technology described in this study demonstrates novel hNSC suspension scale-up and drug loading technologies that enables delivery to the brain for treating CNS diseases.

## 2. Materials and Methods

### 2.1. hNSC culture and EV collection

The hNSCs were previously described [25] and cultured in proliferation media consisting of 1X ANS^™^ Neural Supplement in AB2^™^ medium (Aruna Bio), supplemented with 2 mM L-glutamine (Gibco), 20 ng/ml bFGF (R&D Systems) and 10 ng/ml leukemia inhibitory factor (LIF) (Millipore). To form neurospheres, hNSCs were seeded at 1×10^6^ cells/ml in proliferation media in 1L spinner flasks (Corning) on magnetic stirrer plates at 37° C in 5% CO_2_. Media was collected daily from the spinner flasks for EV isolation for 30 days. Approximately 100L of media was processed for EV collection and concentrated by utilizing tangential flow filtration and anion exchange chromatography.

### 2.2. Nanoparticle Tracking Analysis, Leprechaun analysis and Nanoanalyzer analysis

EV size and concentration were determined using a Nanosight NS300 (Malvern Panalytical), according to manufacturer instructions. Briefly, samples were diluted to a range between 1×10^8^ – 1×10^9^ particles/ml, such that the particles/frame were in the range of 20–60. Five captures were taken over 60 seconds with an infusion rate of 25. From the merged data, the particle concentration (particles/ml) and mean and mode diameter (nm) were acquired.

CD63 levels following EV collection were determined using the Leprechaun (Unchained Labs) Exosome Human Tetraspanin Kit, according to manufacturer instructions. Briefly, 5×10^7^ particles/ml were suspended in solution A and pipetted into the center of the Luni, sealed and incubated overnight. The following day, the Luni was washed in a CW-100 plate washer and fluorescent antibody solution (containing Anti-CD63) was prepared and incubated with sample for 1.5 hours. Following additional washing steps, the chips were scanned on the Leprechaun.

The Flow NanoAnalyzer (NanoFCM) was used to determine the levels of Dox incorporation into EVs. Following Dox loading, EVs were diluted to 1×10^8^/ml in PBS and analyzed on the NanoAnalyzer and compared to unloaded EVs. Excitation/emission for Dox was evaluated using the PE channel and triggered from the SS.

### 2.3. Transmission Electron Microscopy

Negative staining for EVs (1×10^11^ EVs/ml) were performed using a formvar-coated EM grid and treated with phophotunstic acid and analyzed on the Talos L120C 120kV microscope (ThermoFisher).

### 2.4. EV-Dox loading and release

Doxourubicin (Dox) was loaded into hNSC-EVs by passive incubation under alkaline conditions. The large-scale loading by passive incubation was performed as follows: 6×10^13^ EVs were mixed with Dox to a final concentration 100 μM in 200 ml (3×10^11^ EVs/ml). The pH of the suspension was adjusted to 9.5 and was shaken for 1 hour at room temperature and then subsequently neutralized with HCl to return the sample to a pH of 7.0. The EVs were next subjected to ultracentrifugation (100,000 × g) for 1 hour. EV-Dox was washed in PBS and re-concentrated by ultracentrifugation (100,000 × g) for 1 hour. The loading levels of Dox were determined by fluorescence quantification (ex/em: 470/595) on a plate reader (Molecular Devices) and by mass spectrometry by comparing EV-Dox levels to a Dox standard curve.

EV-Dox release assays were performed by performing dialysis using 20K MWCO slide-a-lyzer MINI dialysis devices (Thermo Scientific). EV-Dox samples (100 μL) were dialyzed against 1L of PBS and 10 μL samples were collected at indicated time points. Dox levels were measured by fluorescence quantification on a plate reader, as described above.

### 2.5. Mass Spectrometry

Chemicals and reagents: The stable deuterated isotope labeled internal standard (IS), paclitaxel-d5, was purchased from Cayman Chemical Co. (Ann Arbor, Michigan, USA). Paclixtaxel-d5 was not loaded into EVs and used only as an internal instrument standard as previously described [35]. Ammonium formate, formic acid, acetonitrile (ACN), methanol (MeOH) and water were all liquid chromatography-mass spectrometry (LC-MS) grade reagents from Sigma Aldrich Inc. (St. Louis, MO, USA).

LC-MS/MS conditions: The LC-MS/MS analysis was performed with an ACQUITY ultra-performance liquid chromatography (UPLC) H-Class PLUS system (Waters, Milford, MA, USA) interfaced to a Waters Xevo Micro TQS mass spectrometer with an electrospray ionization (ESI) source (Waters, Milford, MA, USA). Waters Masslynx 4.2 software (Milford, MA, USA) was used for instrumentation and quantitative analysis. The separation was performed on a BDS Hypersil C8 column (50×2.1 mm, 5 μm; Thermo Scientific, West Palm Beach, FL, USA) coupled with a SecurityGuard C8 guard column (4×3.0 mm; Phenomenex, Torrance, CA, USA).

Mobile phase A was 10 mM ammonium formate aqueous buffer with 0.1% formic acid and mobile phase B was MeOH. To separate the analytes, an isocratic elution with a washing gradient was used (time/minute, % mobile phase B): (0.0, 30), (1.5, 30), (4.0, 95), (5.0, 95), (5.1, 30), (6.0, 30). The flow rate was 0.8 mL/min, the column temperature was 40°C, and the autosampler temperature was 20°C. The injection volume was 10 μL and the injection needle was washed with 70% ACN/water (v/v) after each injection. The mass spectrometer was operated in positive electrospray ionization (ESI+) mode. Nitrogen was used as the desolvation gas at a flow rate of 800 L/h and a temperature of 120°C. The cone gas (N2) flow rate was 50 L/h and the source temperature was 120°C. The capillary voltage and the cone voltage were set at 0.5 kV and 50 V, respectively. Argon was used as the collision gas at a collision cell pressure of 3.5×10–3 mbar. Multiple reaction monitoring (MRM) functions were used to detect and quantify the analyte and IS. The collision energy for doxorubicin and paclitaxel-d5 were at 12 eV and 27 eV, respectively. With the dwell time at 0.054 s, the transitions m/z 544.1→397.0 and 881.3→313.0 of the [M + Na] + ions were monitored for doxorubicin and paclitaxel-d5, respectively; while the transitions of m/z 544.1→130.0 and 881.3→591.1 were used for confirmation.

Preparation of stock, standard solutions and samples: The stock solution of paclitaxel-d5 was prepared by dissolving 1.0 mg of solid in 1.0 mL of ACN to yield a concentration of 1.0 mg/mL. Calibration working solutions of doxorubicin were also prepared for the doxorubicin-EV stocks at concentrations of 1, 2, 4, 8, 10, 12, 16, 20, 24, and 30 μg/mL by dilutions from the stock solution. The IS working solution was 1.0 μg/mL paclitaxel-d5 in ACN. Stock solutions were kept at −20°C when not in use.

A volume of 10 μL of the calibration working solution was spiked into 190 μL of brain homogenate or PBS to generate the corresponding calibration standards. The final concentrations of the calibration standards were 0.1, 0.2, 0.4, 1.0, 3.0, 5.0, 8.0, 10.0, 12.0, and 15.0 ng/mL in brain homogenate. The final concentrations of the calibration standards were 0.05, 0.1, 0.2, 0.4, 0.5, 0.6, 0.8, 1.0, 1.2, and 1.5 μg/mL in PBS. Calibration samples were then processed as described below. All biological samples were stored at −80°C when not in use.

A protein precipitation (PPT) method was used for sample preparation. To each 100 μL of biological sample or calibration standard, 1 mL of chilled ACN containing the IS (4.3 ng/mL) were added. The mixture was vortexed with a VX-2500 Multi Tube Vortexer (VWR, Radnor, PA, USA) for 10 min and then centrifuged with an Eppendorf Centrifuge 5427 R (Hamburg, Germany) at 25,001 g and 18°C for 12 min to extract doxorubicin and the IS. To a MS vial, 90 μL of the supernatant and 210 μL water were added to resemble the initial mobile phase composition (30% MeOH/water (v/v)). The samples were vortexed for 10 min before LC-MS/MS analysis.

### 2.6. Caspase and MTS analysis

CT-2A mouse glioma cells were seeded at 20,000 cells/well in a 96-well plate for caspase and MTS assays. After 24 hours, cells were treated with EV-Dox or control conditions for 24 hours and assayed for cytoxicity (MTS assay, Abcam) or caspase 3/7 activation using the Caspase-Glo 3/7 Assay kit (Promega), according to manufacturer guidelines.

### 2.7. Animal studies.

C57BL/6 mice were intravenously injected by tail-vein with hNSC-EV-Dox or Dox only at 1 mg/kg. After 1 hour, brains were perfused, collected, weighed and stored at −80°C. Brain homogenates from the left hemisphere were prepared by resuspending tissue samples in molecular grade water with a beadmill homogenizer at 25 Hz for 45 sec. per cycle for 10 cycles. Homogenates were re-frozen and later processed for mass spectrometry analysis.

### 2.8. Statistical Analyses.

All statistical analyses were performed using Graphpad Prism using either a Student T-test or one-way ANOVA with p-values, as indicated. Statistical analyses were performed on independent replicates.

## 3. Results

### 3.1. Suspension culture of hNSCs results in increased EV production and purity.

**Figure 1:**
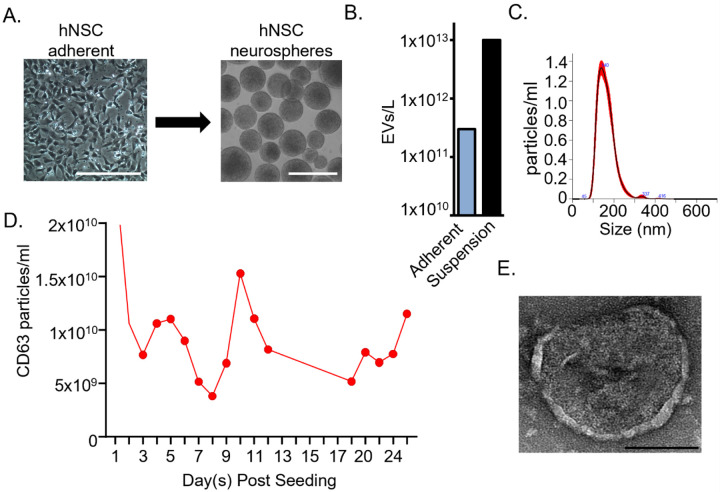
Scale-up culture of hNSC-derived neurospheres and EV production characterization. (**A**) Representative images of hNSCs following one week of neurosphere development, bar: 500 mm. (**B**) Quantification of hNSC-EV production from adherent or suspension culture using Nanoparticle Tracking Analysis. (**C**) Size analysis from NTA of the hNSC-EVs. (**D**) CD63 exhibited consistent levels on suspension hNSC-EVs tracked over time. (**E**) TEM analysis of hNSC-EVs, bar: 100 nm.

To establish a scalable platform amenable to large bioreactors for EV production from adherent hNSCs, the hNSCs were grown in proliferation media in suspension culture in spinner flasks. The hNSCs were able to be grown for more than 30 days without any significant loss in cell viability as determined by sphere morphology (Fig. 1A). Interestingly, the transition from adherent to suspension culture resulted in > 10-fold increase in the EV output (Fig. 1B), as also indicated for other cell types [36]. The EV mean diameter, as determined by nanoparticle tracking analysis (NTA), peaked at 140 nM, consistent with our analysis from adherent cultures (Fig. 1C) [18, 28–31]. To evaluate day-to-day levels in EV production from the suspension culture, CD63 Tetraspanin levels were evaluated on the Leprachaun over time (Fig. 1D). CD63 levels were largely stable throughout the time course, indicating consistent EV output from the spheres. To further characterize the hNSC-EVs, transmission electron microscopy was performed (Fig. 1E). The EVs produced exhibited the typical EV morphology consisting of a lipid membrane surrounding a lumen. Overall, these data demonstrate the hNSCs can be grown in suspension culture for scale-up requirements with improved concentration facilitating their use as a therapeutic.

### 3.2. Loading and quantification of Doxorubicin into hNSC-EVs

As a scalable system for producing EVs from hNSCs was successfully established and characterized (Figs. 1), the potential function of the EVs as a drug delivery vehicle was evaluated. As a proof of concept, doxorubicin (Dox) was chosen as the drug of choice to evaluate for hNSC-EV drug loading and delivery due to several attributes. First, Dox has been demonstrated both as a free-drug or following encapsulation into liposomes (Doxil or Myocet) to have little to no ability to penetrate the blood-brain barrier [37]. Second, Dox is fluorescent and therefore enables easy visualization for both drug loading and cell uptake. Third, Dox has potential to serve as a therapeutic for brain cancers if sufficient brain delivery can be achieved [38–40]. As such, clinical trials are currently ongoing that evaluate methods of focused ultrasound to disrupt the BBB with Dox treatment (clinical trial: NCT05615623).

**Figure 2:**
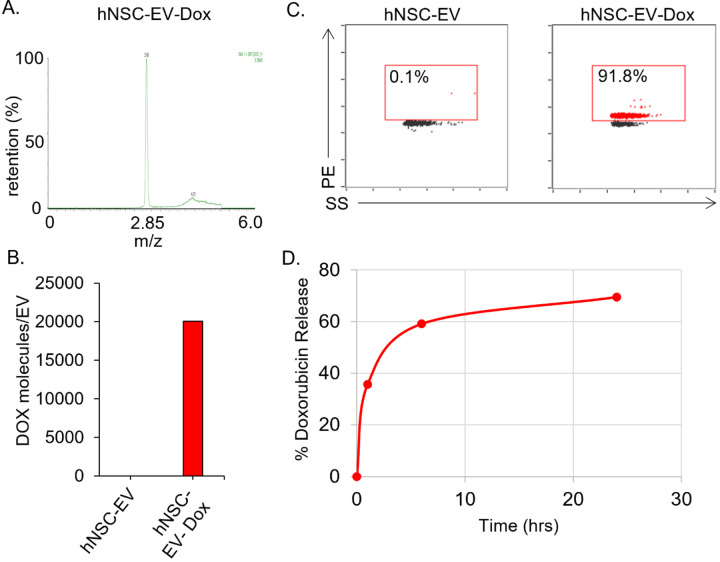
Loading and drug-release evaluation of hNSC-EV-Dox. (**A**). Mass spectrometry analysis of hNSC-EV-Dox indicating main peak for Dox presence. (**B**) Concentration levels expressed as Dox molecules per EV based on mass spec analysis compared to a standard curve. (**C**). Single particle analysis of hNSC-EV samples indicating loading levels of Dox. (**D**) Dox-release assay performed by dialysis and sampling after 1, 6 and 24 hours, with concentrations determined by fluorescence measurements (ex/em: 470nm/595nm) compared to starting concentrations.

To load Dox into the hNSC-EVs, passive incubation was performed for 1 hour at room temperature under alkaline conditions at a pH of 9.5. This pH enables free Dox to be deprotonated, as the isolectric point is 8.4, and was hypothesized to enable Dox to freely pass across EV membranes. After the incubation, the EV-Dox sample was neutralized and returned to a physiological pH, promoting Dox entrapment into EVs. To accurately measure Dox in the EVs and then later in brain tissue, a mass spectrometry protocol was developed using previously established protocols [41]. Following the analysis by mass spectrometry, a single peak was observed pertaining to Dox in the EVs (Fig. 2A). Using a standard curve of free Dox, the number of molecules per EV was calculated to be approximately 20,000 (Fig. 2B). No molecules were detected in unloaded EVs. To further evaluate the Dox loading levels of hNSC-EVs, nano-flow cytometry was performed (Fig. 2C). More than 90% of the hNSC-EV were successfully loaded with Dox, indicating the high efficacy of alkaline-based passive loading. Next, to evaluate how quickly Dox is released from the EVs, dialysis of EV-Dox was performed, and Dox levels were measured by fluorescence over time (Fig. 2D). Approximately 35% of Dox was released in 1 hour and 70% of the Dox was released from the EVs following 24 hours. These data demonstrate that Dox can be efficiently loaded into hNSC-EVs and suggest that it will be released upon uptake into cells.

### 3.3. Dox-loaded hNSC-EVs promote cell death in glioma cells

**Figure 3:**
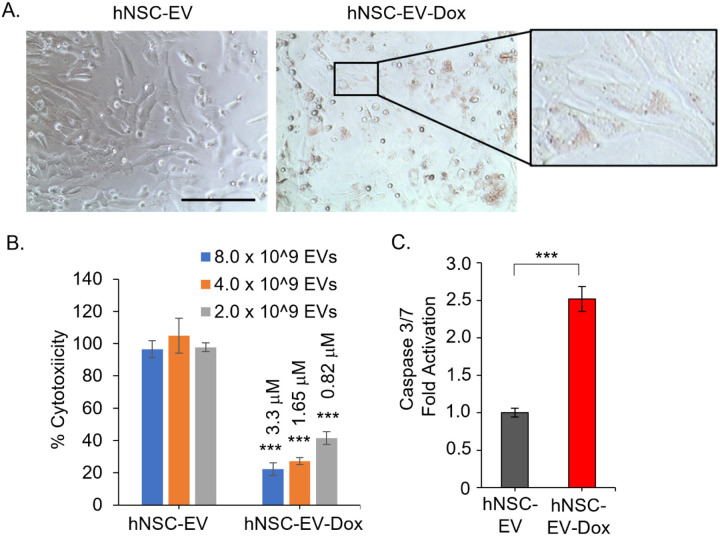
hNSC-EV-Dox promotes cell death in glioma cells. (**A**) Phase-contrast images of CT-2A mouse glioma cells treated with equal concentrations of hNSC-EVs or hNSC-EV-Dox for 24 hours, bar: 200 mm. (**B**) Cytotoxicity levels performed by MTS assay in CT-2A cells after 48 hours of treatment with hNSC-EV or hNSC-EV-Dox at indicated concentrations, n=3 per group, ***p < 0.001, using one-way ANOVA with Tukey’s post analysis, comparing hNSC-EV to hNSC-EV-Dox at dose-matched concentrations. (**C**) Caspase 3/7 activity assay following 24 hours treatment of CT-2A cells with with hNSC-EV or hNSC-EV-Dox at 3.3 mM, n=3 per group, ***p < 0.001 using Student T-test.

To evaluate if EV-Dox will promote cytotoxicity in glioma cells, CT-2A cells were treated with Dox-loaded EV and compared to EVs alone (Fig. 3). After 24 hours of treatment, Dox uptake could be visualized by light microscopy in the cells (Fig. 3A), along with a clear reduction in cell confluency. To evaluate the cytotoxic effects of hNSC-EV-Dox in mouse glioma cells, MTS assays were performed with increasing concentrations of hNSC-EVs (Fig. 3B). A dose-dependent reduction in cell viability and proliferation was observed from the hNSC-EV-Dox treatment (Fig. 3B). Next, apoptotic induction was measured using caspase 3/7 activity assays following the treatment of hNSC-EVs or hNSC-EV-Dox samples for 24 hours (Fig. 3C). A ~ 2-fold increase in caspase activity was observed from the hNSC-EV-Dox treatment, compared to the hNSC-EV alone, indicating that the hNSC-EV-Dox samples activated apoptotic cascade signaling within the cells. Altogether, these data demonstrate that Dox-loaded hNSC-EVs are readily taken up by glioma cells, and this subsequently results in their loss of proliferation and promotion of apoptotic pathways.

### 3.4. Dox-loaded hNSC-EVs are transported to the brain

**Figure 4:**
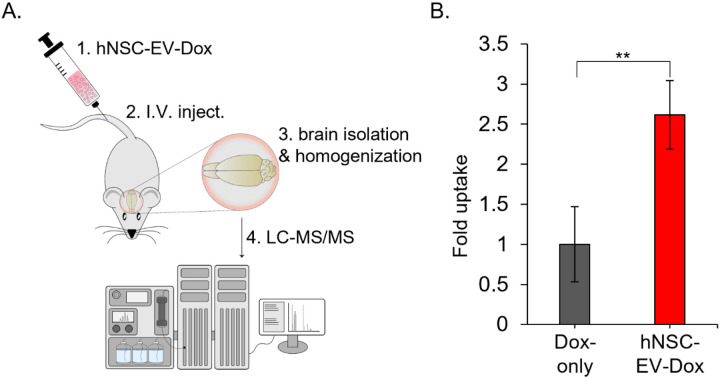
hNSC-EV-Dox have increased brain uptake compared to Dox alone. (**A**) Method for evaluating brain uptake by hNSC-EV-Dox. Following hNSC-EV-Dox loading, mice were intravenously (I.V.) injected with 1 mg/kg of Dox alone or hNSC-EV-dox for 1 hour and brains were isolated and homogenized for mass spectrometry, n=3 mice per group. (**B**) Quantification of Dox levels by LC-MS/MS in mouse brain tissue of Dox alone or hNSC-EV-Dox, **p < 0.01 using Student T-Test.

Chemotherapeutic drugs, such as Dox, often fail to penetrate the BBB and therefore have limited utility for treating brain cancers like glioblastoma [37]. The hNSC-EVs were previously demonstrated to penetrate the BBB and have therapeutic efficacy for treating stroke and traumatic brain injury [18, 28–31]. As the hNSC-EVs were successfully loaded with Dox and had efficacy at inhibiting glioma cell proliferation and promoting apoptosis *in vitro*, we next sought to determine if hNSC-EVs could efficiently deliver Dox through the BBB to the brain. Our previous studies demonstrated that hNSC-EVs can be detected in the rodent brain at 1 hour, but not 24 hours, post injection by single photon emission computed tomography, so we evaluated Dox levels in the brain at 1 hour [18]. Mice were injected intravenously with hNSC-EV-Dox or Dox-only at equivalent concentrations as determined by mass spectrometry. At 1 hour post EV injection, the mice were sacrificed and brain tissues were perfused and homogenized for mass spectrometry analysis of Dox to determine brain tissue drug concentrations (Fig. 4). Importantly, mice treated with hNSC-EV-Dox had an approximate 2.5-fold increase over mice treated with Dox alone. These data indicate that the hNSC-EVs deliver Dox through the BBB and may serve as a useful therapeutic for brain cancers. Moreover, these studies demonstrate the utility of hNSC-EVs for drug delivery in CNS diseases.

## 4. Discussion

Drug Delivery across the BBB for CNS diseases remains a significant challenge in drug development [2]. In this study, hNSC-EVs were evaluated as a drug-delivery vehicle for drugs that have little-to-no BBB penetration. As a proof of concept, Dox was chosen as the drug of choice to evaluate due to its low BBB penetrance [37], previous studies indicating EV-Dox loading ability [42–47], and the potential utility of doxorubicin for brain cancer treatment if BBB penetrance is overcome [38–40].

A major hurdle in the extracellular vesicle field that hampers their therapeutic use is scale-up requirements. To overcome this issue, a suspension culture system using neurospheres from the hNSCs was developed. This system provided several benefits over the traditional adherent culture system, which included (1) cost-reduction due to the removal of basement membrane protein (i.e. laminin) vessel coating, (2) increased yield based on total particles collected. This suspension system enhances the use of native hNSC-EVs for therapeutic treatment in stroke and TBI [18, 29–31], and for further development in drug delivery of small molecules, RNAs or proteins [28].

Scale-up approaches for other stem cell types, such as MSC or induced pluripotent stem cells (iPSC) that make use of bioreactors have been previously described [32–34]. While EV production from iPSCs grown in bioreactors has not been described, EVs produced from MSCs grown in bioreactors with the aid of microcarriers has been achieved [48]. Neurosphere culture from hNSC for the purpose of generating EVs has also been described [49–51]. Here we expanded upon these findings to show that scale-up culture of hNSC-derived neurospheres can facilitate EV-based drug delivery for CNS diseases.

Following the establishment of a scalable system hNSC-EV production, Dox was loaded into hNSC-EVs at a large scale (~ 6.0×10^13^ hNSC-EVs). While previous studies have utilized sonication or electroporation for Dox-loading of EVs [43–46], these approaches are often not scalable for therapeutic applications beyond proof of concept and could potentially damage EV integrity. As such, we focused on optimizing co-incubation under alkaline conditions as a passive loading method, which was found to be equivalent or exceed the membrane-based disruption approaches, as previously described [42]. Indeed, by single EV assessment, we found that Dox-loading levels could be achieved above 90%, with approximately 20,000 molecules/EV, which demonstrates the utility of the alkaline loading approach. Importantly, these levels provide a clinically-feasible dose concentration for the treatment of brain cancer. To our knowledge, this is the first example of using a mild alkaline pH to load doxorubicin into EVs. However, previous studies have utilized stronger alkaline conditions (pH > 11), in combination with sonication, on cells as a method to generate EV-mimetics for the purpose of loading drugs, such as dexamethasone [52]. The approach described is considerably different, as it is used with native EVs, not on cells to generate EV-mimetics, and does not rely on disrupting EV membranes.

We evaluated Dox release from EVs by dialysis and found that after 1 hour, > 60% was still retained within the EVs. This suggests that some Dox may be retained within the EVs as it is transported through the BBB to target cells of interest. We next confirmed the *in vitro* efficacy of hNSC-EV-Dox at promoting cell death using a glioma cell line. Lastly, we demonstrated that hNSC-EV-Dox enhances the BBB penetrance of Dox into the brain. These findings establish hNSC-EV-Dox as a potential treatment for brain cancers, such as glioblastoma.

As a therapeutic, Dox suffers from several drawbacks beyond BBB penetrance, such as off-target toxicity. The formulation of Dox into pro-drug conjugates, such as liposomes or other modifications, have been suggested to reduce these drawbacks and improve pharmacokinetics, reduce Dox resistance in tumors and provide opportunities for oral route of administrations [53, 54]. Interestingly, Dox-loaded EVs have also been shown to have reduced cardiotoxicity compared to Dox alone [45]. The loading of pro-drug formulations of Dox into EVs may therefore provide additional benefits, such as reducing tumor resistance, for cancer treatments.

The development of scalable, Dox-loaded hNSC-derived EVs demonstrates an enabling technology for hNSC-derived EV drug delivery. The use of hNSCs as an EV producer cell could potentially be expanded for the delivery of other cargo, including proteins or siRNA through endogenous genetic engineering approaches as we have demonstrated previously [55] or by exogenous loading into EVs directly [14]. Additional studies are warranted to expand the drug delivery utility of hNSC-EVs.

## 5. Conclusion

The present study focused on developing a scalable neurosphere suspension system for producing hNSC-EVs and demonstrating the capability for delivery of the CNS impermeable drug, doxorubicin, to the brain. This proof-of-concept study establishes hNSC-EVs as a drug-delivery vehicle, which can be broadly applied to most CNS diseases. Furthermore, the novel alkaline passive EV-loading method described here may have broad utility for basic compounds to facilitate entrapment for drug delivery purposes. Additional studies will expand the drug-delivery capabilities of hNSC-EVs for other BBB impermeable chemotherapeutics such as Paclitaxel and Gleevec. Future evaluation of hNSC-EV loaded chemotherapeutics in GBM animal models will enable these drugs for future clinical development.

## Highlights

- Scalable production of hNSC-derived EVs in a suspension culture.
- High efficiency alkaline passive loading of Dox into hNSC-EVs.
- Dox release from hNSC-EVs drives glioma cell cytotoxicity.
- hNSC-derived EVs enable drug delivery through the BBB to the brain.
